# Exceptional response to PD-1 inhibition immunotherapy in advanced metastatic osteosarcoma with tumor site infection

**DOI:** 10.1136/jitc-2022-004673

**Published:** 2022-09-09

**Authors:** Meng Li, Qiyuan Bao, Zhusheng Zhang, Beichen Wang, Zhuochao Liu, Junxiang Wen, Rong Wan, Yuhui Shen, Weibin Zhang

**Affiliations:** 1 Department of Orthopaedics, Ruijin Hospital, Shanghai Jiao Tong University School of Medicine, Shanghai, China; 2 Shanghai Key Laboratory for Bone and Joint Diseases, Shanghai Institute of Traumatology and Orthopedics, Shanghai, China

**Keywords:** Immunotherapy, Case Reports, Sarcoma

## Abstract

Recent clinical trials have demonstrated a lack of activity of immune checkpoint inhibitors (ICIs) against osteosarcoma. Previous clinical observations have demonstrated a potential immune-stimulatory effect of tumor site infection for osteosarcoma patients. However, whether such infection could augment the efficacy of immunotherapy such as ICIs is currently unknown. Here we report a case of a heavily pretreated 14-year-old boy with pulmonary metastatic osteosarcoma, who has suffered from multiple wound infections and thoracic empyema after previous metastasectomy. Despite the ongoing tumor site infection, the patient had a rapid and durable (11 months) remission of the metastatic lesions after the administration of the Programmed cell death-1(PD-1) inhibitor camrelizumab. No serious ICI-related toxicities or worsening of the infection were noticed during the treatment. Correlative analysis suggested that intratumoral CD8+ T cell infiltration, Programmed death-ligand 1(PD-L1) expression and IFN-γ expression were increased in the tumor microenvironment postinfection versus preinfection. Furthermore, using RNA-seq gene expression analysis, we found a variety of checkpoint targets were also upregulated such as *CD200*, *TIGIT*, *LAG3*, etc. Our report supports the hypothesis of tumor site infection as a potential synergistic mechanism in the tumor microenvironment for ICI immunotherapy.

## Background

Osteosarcoma (OS) is a highly metastasizing bone tumor that mainly affects children and young adolescents. The prognosis of metastatic OS remains dismal, with a 5-year overall survival rate of only 20%–30%.^1^ Hence, new treatment approaches are urgently needed to improve the clinical outcomes for these patients. In recent years, immune checkpoint inhibitors (ICIs) have emerged as a significant breakthrough in immunotherapy for various epithelial cancers. However, current clinical trials have suggested a lack of activity of ICIs against OS. In the SARC028 trial, 1/22 OS patients achieved partial response, with a median progression-free survival (PFS) of 8 weeks.^2^ In the PEMBROSARC study, only 1/17 OS patients reached Partial Response(PR), and the median PFS was 1.4 months.^3^ The other clinical trials of ICI monotherapy reported an even worse outcome, with no objective response seen in OS patients (online supplemental table 1). To date, the mechanism underlying the resistance of OS to immunotherapy remains still unclear. Some research demonstrated that OS is an immunologically cold tumor, characterized by poor infiltration of immune cells, low activity of intratumor T cells and the lack of tumor neoantigens.^4^

10.1136/jitc-2022-004673.supp1 Supplementary data

Paradoxically, OS may be one of the earliest reported cases of targeting cancer by manipulating the tumor microenvironment for therapeutic purposes. In 1891, William Coley treated bone sarcoma by introducing bacterial infections around the tumor site, which is now regarded as a prototype of immunotherapy. Since then, multiple clinical observations have demonstrated a potential immune-stimulatory effect of tumor site infection in OS.^5 6^ However, whether such infection is a therapeutic contraindication or antitumor synergistic factor to present-day cancer immunotherapy such as ICIs is currently unknown.

Here we report the case of a heavily pretreated 14-year-old boy with pulmonary metastatic OS, who has suffered from multiple wound infections and thoracic empyema after the metastasectomy. Despite the ongoing tumor site infection, the patient had a rapid and durable remission of the metastatic lesions after the administration of the PD-1 inhibitor camrelizumab. No serious ICI-related toxicities or worsening of the infection were noticed during the treatment. Correlative analysis of tumor specimens preinfection and postinfection supports the hypothesis of tumor site infection as a synergistic mechanism in the tumor microenvironment for ICI therapy.

## Case presentation

**Figure 1:**
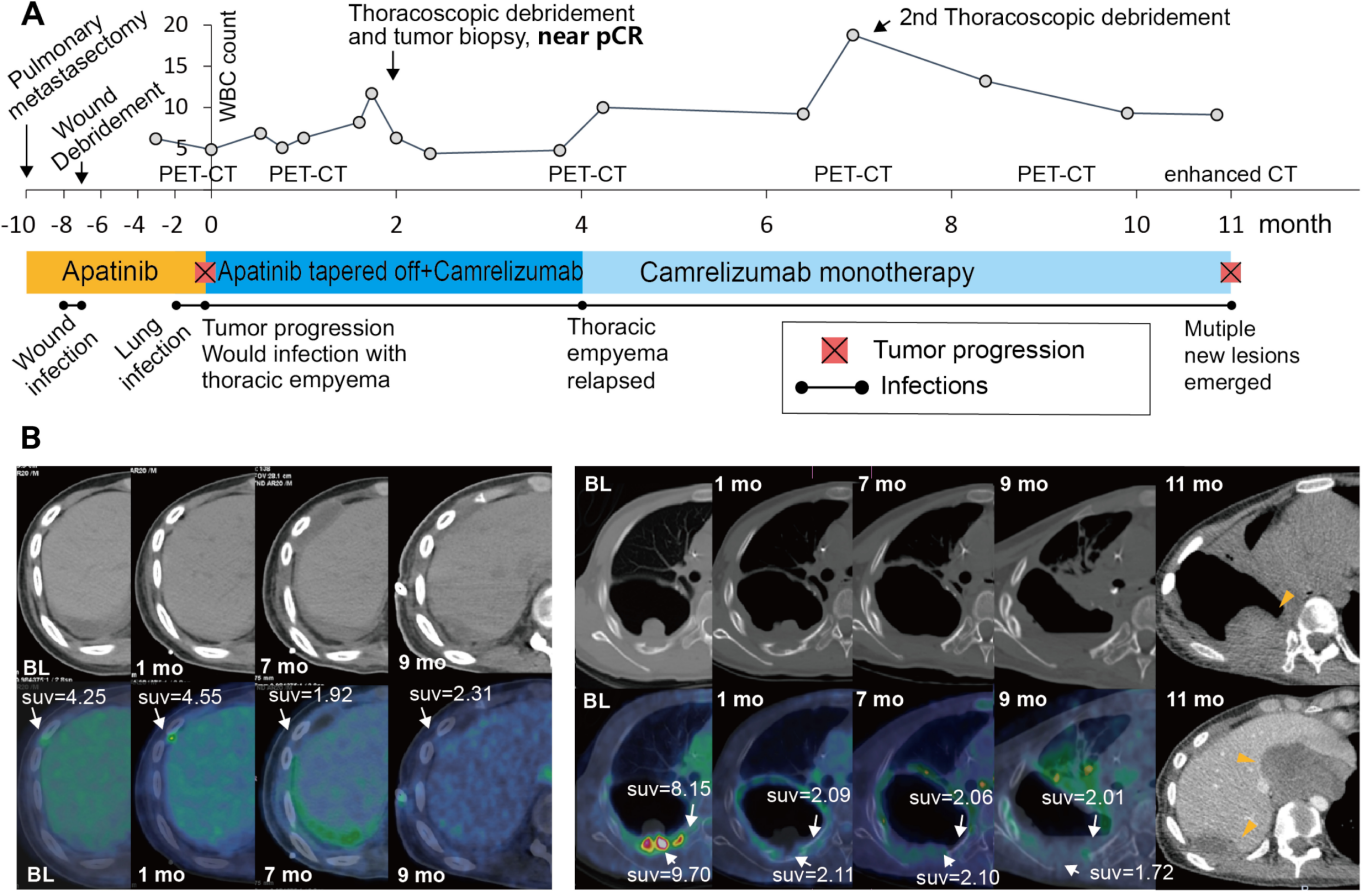
The timeline of the patient’s infection, immunotherapy and oncological outcomes. (A) The patient has previously developed wound infection and lung infection, and had ongoing wound infection with thoracic empyema when initiating the PD-1 inhibitor Camrelizumab. The change of the serum white blood cell (WBC) count and 3 surgical debridement procedures during the course of the immunotherapy are denoted. Tumor biopsy during the thoracoscopic debridement (2 months post-immunotherapy) suggested a pathological near-complete response of the tumor. (B) The FDG PET/CT images of the metastatic lesions before and during the course of the immunotherapy. The pleural and costal metastases demonstrated a rapid and durable response to Camrelizumab (white arrow). Enhanced CT scans at 11 months post-treatment suggested pleural tumor relapse (yellow arrowhead) as well as multiple new lesions in the mediastinal (yellow arrowhead), pleural (yellow arrowhead) and inferior vena cava (not shown) regions.

A 14-year-old male patient with metastatic OS originating from the right tibia visited our clinics due to dyspnea in June 2019. A chest CT scan demonstrated a 14 cm pulmonary metastatic lesion of the right lower lobe despite two prior lines of chemotherapy. He underwent pulmonary metastasectomy with marginal resection, and the postoperative pathology confirmed the diagnosis OS metastasis with negative PD-L1 expression and microsatellite stable status. He then received 10 months of antiangiogenic therapy with apatinib, during which wound infection and lung infection occurred (figure 1A). In April 2020, he developed pleural and costal recurrence, which were progressive and 18F-fluorodeoxyglucose (18F-FDG)-avid on PET/CT, complicated by another wound infection and thoracic empyema (figure 1B). Soon after chest tube drainage and intravenous antibiotics, the patient’s general condition improved with minimal systemic symptoms reported. Therefore, in consideration of the progressive disease and the risk of worsening wound infection due to the antiangiogenic agent, the multidisciplinary oncology team decided to taper off apatinib during the following 4 months to avoid the potential tumor rapid rebound and start camrelizumab (a monoclonal programmed death-1 (PD-1) antibody) 200 mg once every 2 weeks, followed by surgical debridement procedures if necessary. With a close surveillance of the infection, we surprisingly observed a remarkable radiologic response of the recurrent lesions, as well as a pathologic near-complete response in the tumor biopsy during thoracoscopic debridement (figure 1B).

There were no serious toxicities associated with PD-1 inhibition therapy during treatment despite the ongoing infection. We found no obvious aggravation of the infection following PD-1 blockade, as demonstrated by the white cell count (figure 1A) and the neutrophil percentage. Side effects such as acute pneumonitis and cytokine release syndrome were not observed in this patient. The metastatic lesions demonstrated a drastically reduced 18F-FDG uptake and stabilized for a total of 11 months until tumor progression, which was confirmed by enhanced CT showing mediastinal mass, inferior vena cava tumor thrombus and pleural recurrence as new lesions in March 2021 (figure 1B). The patient then developed anorexia and cachexia due to mediastinal compression. The thoracic infection soon worsened, precluding the opportunity of tumor rebiopsy or further PET scan. After the referral to hospice care, the patient deceased at the end of June 2021.

**Figure 2:**
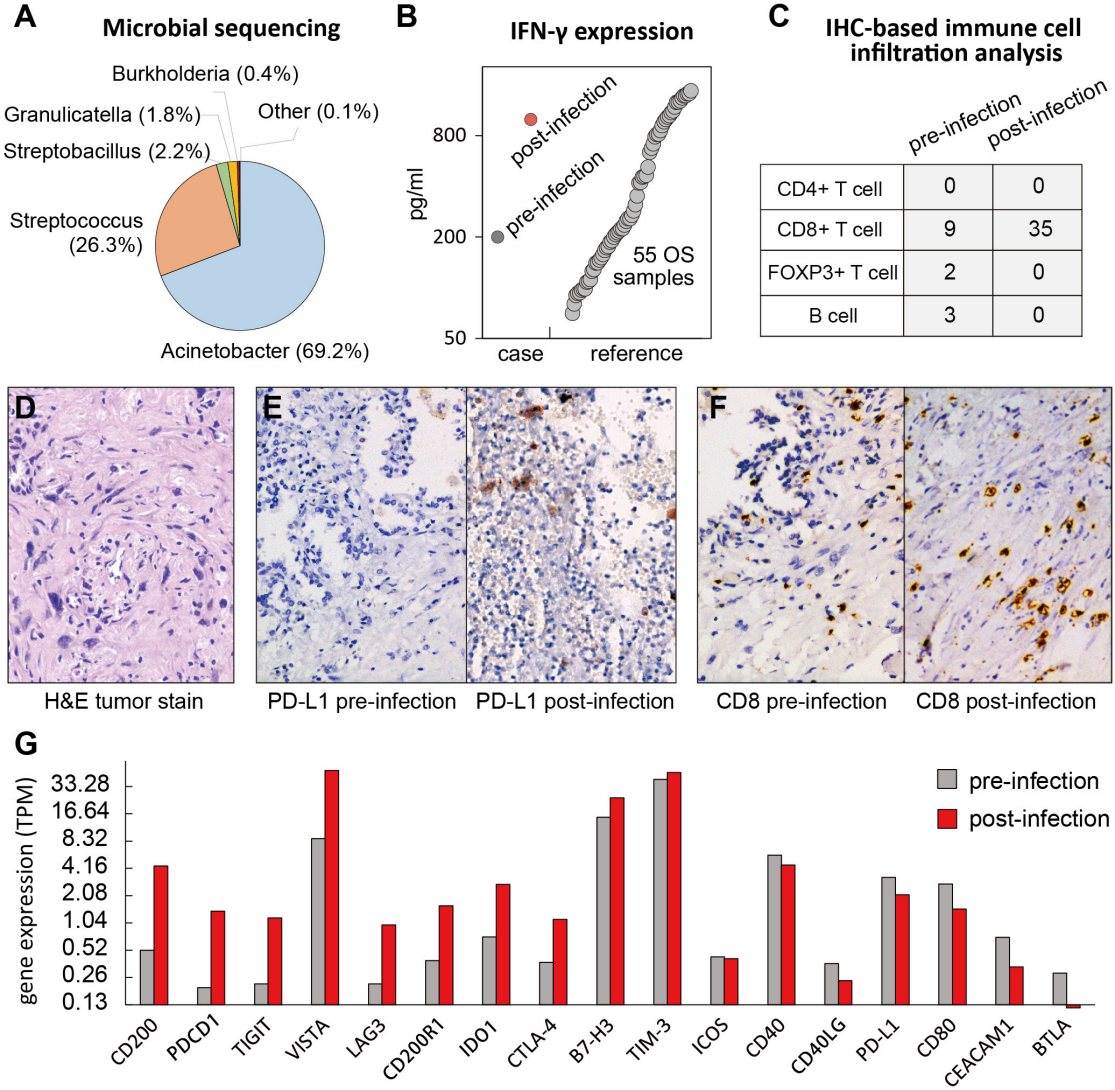
Correlative analysis of the osteosarcoma tumor specimens pre- and post-infection (A) The metagenomic next-generation sequencing (mNGS) of the pleural effusion. Numbers in the bracket indicate the percentage (%) of Genus absolute abundance of the bacteria. No fungus, virus or parasite was detected. (B) The Luminex cytokine immunoassay suggested a 4-fold increased IFN-γ expression in post- versus pre-infection tumor samples. An in-house dataset of fifty-five osteosarcoma samples were also plotted as a reference. (C) Estimation of intra-tumoral immune infiltration by immunohistochemistry suggested higher CD8+ T cell infiltration (cells per high-power field), but not CD4+, FOXP3+ T cell or B cells, in post-infection compared to the pre-infection specimen. (D) The H&E staining of pulmonary metastasectomy sample confirm the diagnosis of recurrent osteosarcoma. (E-F) The immunohistochemical (IHC) staining of PD-L1 and CD8 in the metastatic lesions pre- and post-infection. (G) RNA-seq gene expression of several potential check-point molecular targets in post-infection compared to the pre-infection samples, as indicated as transcript per million (TPM).

To provide translational insight into the potential immune priming effect of tumor site infection to PD-1 therapy, we profiled the metastatic tumor sample preinfection and postinfection (biopsy during thoracoscopic debridement) using whole-exome sequencing, low-coverage whole-genome sequencing and RNA-sequencing(RNA-seq). In addition to the traditional microbial culture, we performed metagenomic next-generation sequencing of the pleural effusion and found a variety of microorganisms at the tumor site, including Acinetobacter (69.2%), Streptococcus (26.3%) (also reported by William Coley) and Streptobacillus (2.2%), etc (figure 2A and online supplemental table 2). We did not observe any previously reported genetic predictive biomarker in terms of gene alteration (such as PD-L1 amplification) or total mutation burden high. Furthermore, we performed a cytokine immunoassay (Luminex, Thermo Fisher) to compare the inflammatory factors in the tumor microenvironment (online supplemental figure 1). Surprisingly, we found a fourfold increased IFN-γ expression in postinfection versus preinfection, compared with our in-house reference dataset (figure 2B). The immunohistochemistry staining suggested that the postinfection intratumor region was 1% PD-L1 positive, with a high CD8+ T cell infiltration compared with the preinfection one (figure 2C–F). In contrast, other immune cells, such as B cells, Treg and neutrophils, had minimal changes after the infection (online supplemental table 3). Interestingly, RNA-seq gene expression analysis of postinfection versus preinfection samples revealed an increased expression of *PDCD1*, as well as other well-known immune checkpoint molecules that are potentially targetable, such as *CD200*, *LAG3*, *TIGIT*, *IDO*, *CTLA-4*, etc (figure 2G). The methodological details are shown in online supplemental method.

10.1136/jitc-2022-004673.supp2 Supplementary data

10.1136/jitc-2022-004673.supp4 Supplementary data

10.1136/jitc-2022-004673.supp3 Supplementary data

10.1136/jitc-2022-004673.supp5 Supplementary data

## Discussion

OS is typically considered an ‘immunologically cold tumor’ with minimal PD-1 and PD-L1 expression and the lack of inflammatory immune cell and T cells infiltration, which is a plausible explanation for the observed resistance to immune checkpoint inhibitors. In contrast, tumor site infection has long been hypothesized as an immune activation factor in orthopedic oncology, potentially favorable for patient survival during the postoperative adjuvant setting. However, whether such infection could boost antitumor immunity as a priming effect for ICIs in the refractory and recurrent setting is unknown.

Our report might be the first one supporting such hypothesis since the durable antitumor response of camrelizumab seen in our case far exceeded the outcomes from previous clinical trials of PD-1 inhibition monotherapy (online supplemental table 1). There was no other known predictive biomarker such as microsatellite instability, gene alteration (such as PD-L1 amplification) or TMB in this case, and the tumor response is unlikely due to the infections per se. To further investigate such hypothesis, we demonstrated that tumor site infections could modulate the tumor immune microenvironment at the cellular and molecular levels. These findings are consistent with previous translational studies showing that TLR4 activation by lipopolysaccharide (LPS) triggered CD8+ cells infiltrating into lung metastases and suppressed tumor progression in the mouse model. In vivo depletion of CD8+ T cells exacerbated these antitumor effects of LPS.^7^ Furthermore, Nakane *et al*^8^ reported that introducing *Staphylococcus aureus* to mice could enhance the IFN-γ production, especially in the non-lethal infection setting. However, since the microbial sequencing demonstrated multiple microorganisms in our case, other mechanisms such as immune modulation by metabolites derived from the complex interaction between lung microbiota and host microenvironment remain to be explored.

The safety and tolerability of initiating PD-1 blockade therapy during the ongoing infection for patients with advanced cancer remain another interesting question. Conventionally, the National Comprehensive Cancer Network (NCCN) guideline has recommended screening for any autoimmune disease, endocrinopathy and infectious disease with caution before ICI treatment. Some reports suspect that blocking the PD-1/PD-L1 axis may disrupt the immune control of specific opportunistic infections such as tuberculosis.^9^ However, the positive role of PD-1 blockades in treating acute and chronic infectious disease has recently emerged to gain interest, given that PD-1 pathway might permit the microorganisms to escape the elimination by the host’s immune system, especially for the chronic infection.^10^ In our case, we initiated ICI at the stage when the ongoing infection was controlled and tolerated with no evidence of sepsis or systemic inflammatory response syndrome. Close surveillance with a multidisciplinary team was prepared during the course of ICI for the patients. Our results indicated that an ongoing infection might, at least in our case, not be a contraindication for the use of PD-1 inhibition therapy.

## Conclusion

We described a patient with refractory pulmonary metastases of OS, who has suffered from ongoing infection and demonstrated a rapid and durable remission of the metastatic lesions after the administration of the PD-1 inhibitor. Our report highlights the importance of rediscovering the microbial and the host mechanism of tumor site infection as a synergistic effect for ICI in relapsed and refractory OS.
