# Supplementary material for: Exceptional response to PD-1 inhibition immunotherapy in advanced metastatic osteosarcoma with tumor site infection
Source: J Immunother Cancer. 2022 Sep 9;10(9):e004673. doi: 10.1136/jitc-2022-004673 (PMC9472102; doi:10.1136/jitc-2022-004673)
Supplement: Supplementary data [file jitc-2022-004673supp001.pdf]

Supplementary Table 1 Summary of Case Reports and Clinical trials of anti-PD1/PD-L1 in Advanced Osteosarcoma

Author（year published）	Therapy	Disease	Type	result
Merchant[1]（2016）	Ipilimumab	recurrent or refractory solid tumors, including osteosarcoma (n=8)	multicentre, open-label, single-arm, phase 1-2 trial	no CR or PR
Tawbi[2](2017)	pembrolizumab	advanced soft tissue and bone sarcomas	Multicentre, Single arm, Phase 2 Trial	1/22 PR in osteosarcoma, the mPFS and the mOS was 8 weeks [95% CI, 7-9] and 52 weeks [95% CI, 40-72]in BS
D'Angelo [3](2018)	Nivolumab +/- ipilimumab	Metastatic or unresectable sarcoma (incloud 9 bone sarcomas)	non-comparative multi-center randomized phase II study	On the nivolumab monotherapy arm, The mPFS was 1.7 months [n=42, 95% CI (1.4–4.3months). The mOS was 10.7months [n=42, 95% CI (5.5–15.4)]， The longest PFS and OS was 13months and 16.5 months,
Le Cesne[4](2019)	pembrolizumab +cyclophosphamide	metastatic and/or unresectable osteosarcoma	open-label, multicentre, phase 2 study	1/17PR，The 6month non-progression rate was 13.3% (95% CI = 1.7-40.5). The mPFS was 1.4 months (95% CI = 1.0 months-1.4 months), and the mOS was 5.6 months (95% CI = 2.1 months-12.1 months)，
Davis[5] (2020)	Nivolumab	the common paediatric solid tumours	multicentre, open-label, single-arm, phase 1-2 trial	no significant single-agent activity
xie[6]（2020）	Apatinib +camrelizumab	Advanced osteosarcoma	single- arm, open- label, phase 2 trial	The mPFS of 6.2 months (95% CI 4.0 to 6.9) and CBR of 30.2% (95% CI 17.2% to 40.1%)
Geoerger[7] (2020)	Atezolizumab	solid tumours	multicentre phase 1–2 study	no CR or PR in osteosarcoma patients(n=10)
Boye[8] (2021)	pembrolizumab	Advanced osteosarcoma	single-arm, open-label, phase 2 trial	Estimated mPFS was 1.7 months (95% CI 1.2–2.2). mOS was 6.6 months (95% CI 3.8–9.3)， At time of data cut-off, 11 patients were deceased due to osteosarcoma.
Sterz[9] (2021)	Ipilimumab+ nivolumab	metastasized osteosarcoma	Case report	PFS: at least one year

PFS: median progression-free survival. *mOS*: median overall survival. *CR*: complete response. *PR*: partial response *OR*: objective responses
BS: bone sarcoma. *CBR* :the proportion of patients who did not have disease progression at 24 weeks.

Reference

1. Merchant MS, Wright M, Baird K, et al. Phase I Clinical Trial of Ipilimumab in Pediatric Patients with Advanced Solid Tumors. Clin Cancer Res 2016;22(6):1364-70.
2. Tawbi HA, Burgess M, Bolejack V, et al. Pembrolizumab in advanced soft-tissue sarcoma and bone sarcoma (SARC028): a multicentre, two-cohort, single-arm, open-label, phase 2 trial. Lancet Oncol 2017;18(11):1493-501.
3. D'Angelo SP, Mahoney MR, Van Tine BA, et al. Nivolumab with or without ipilimumab

treatment for metastatic sarcoma (Alliance A091401): two open-label, non-comparative, randomised, phase 2 trials. Lancet Oncol 2018;19(3):416-26.

4. Le Cesne A, Marec-Berard P, Blay JY, et al. Programmed cell death 1 (PD-1) targeting in patients with advanced osteosarcomas: results from the PEMBROSARC study. Eur J Cancer 2019;119:151-57.

5. Davis KL, Fox E, Merchant MS, et al. Nivolumab in children and young adults with relapsed or refractory solid tumours or lymphoma (ADVL1412): a multicentre, open-label, single-arm, phase 1-2 trial. Lancet Oncol 2020;21(4):541-50.

6. Xie L, Xu J, Sun X, et al. Apatinib plus camrelizumab (anti-PD1 therapy, SHR-1210) for advanced osteosarcoma (APFAO) progressing after chemotherapy: a single-arm, open-label, phase 2 trial. J Immunother Cancer 2020;8(1).

7. Geoerger B, Zwaan CM, Marshall LV, et al. Atezolizumab for children and young adults with previously treated solid tumours, non-Hodgkin lymphoma, and Hodgkin lymphoma (iMATRIX): a multicentre phase 1-2 study. Lancet Oncol 2020;21(1):134-44.

8. Boye K, Longhi A, Guren T, et al. Pembrolizumab in advanced osteosarcoma: results of a single-arm, open-label, phase 2 trial. Cancer Immunol Immunother 2021;70(9):2617-24.

9. Sterz U, Grube M, Herr W, et al. Case Report: Dual Checkpoint Inhibition in Advanced Metastatic Osteosarcoma Results in Remission of All Tumor Manifestations-A Report of a Stunning Success in a 37-Year-Old Patient. Front Oncol 2021;11:684733.
