# Supplementary figures and images for: Exceptional response to PD-1 inhibition immunotherapy in advanced metastatic osteosarcoma with tumor site infection
Source: J Immunother Cancer. 2022 Sep 9;10(9):e004673. doi: 10.1136/jitc-2022-004673 (PMC9472102; doi:10.1136/jitc-2022-004673)

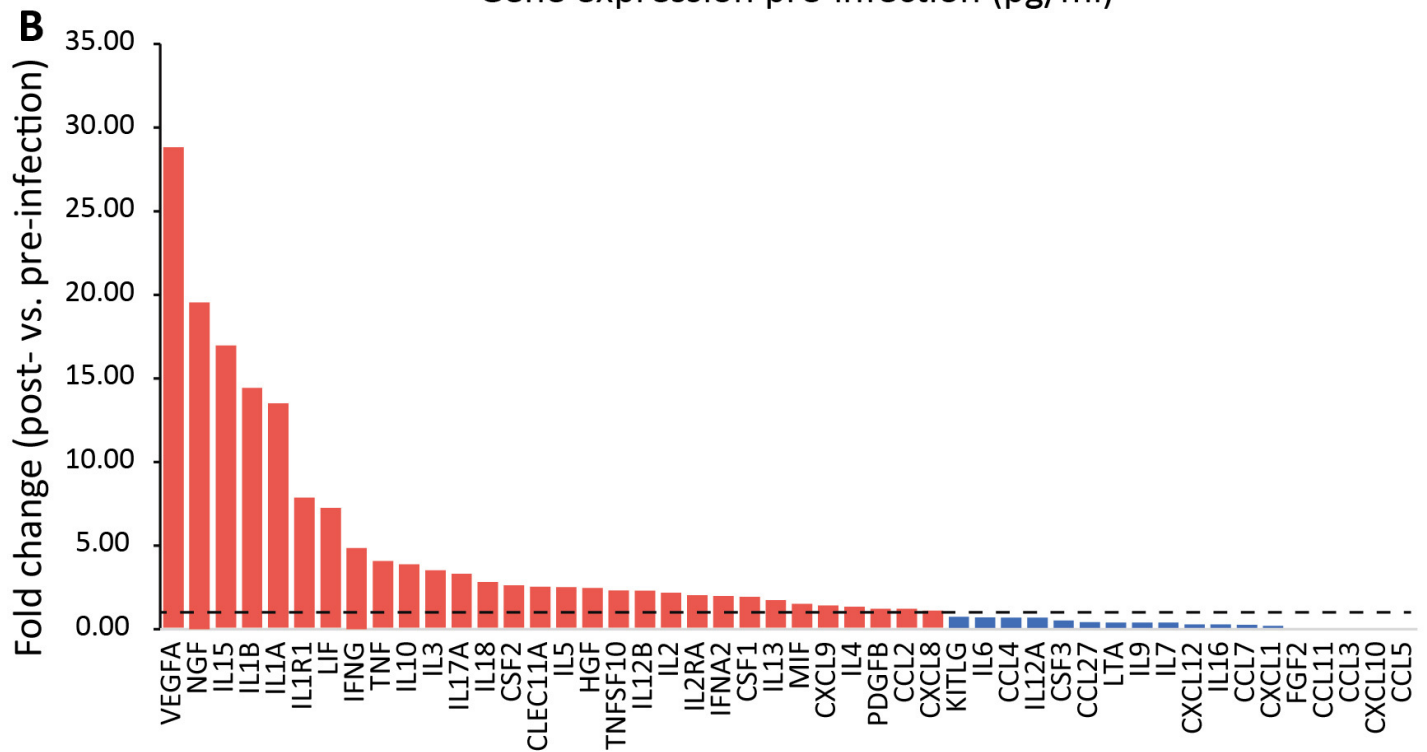

Supplement: Supplementary data [file jitc-2022-004673supp004.pdf]
