# Supplementary material for: Exceptional response to PD-1 inhibition immunotherapy in advanced metastatic osteosarcoma with tumor site infection
Source: J Immunother Cancer. 2022 Sep 9;10(9):e004673. doi: 10.1136/jitc-2022-004673 (PMC9472102; doi:10.1136/jitc-2022-004673)
Supplement: Supplementary data [file jitc-2022-004673supp003.pdf]

Supplementary Table 3 immune infiltration density in pre- and post-infection tumor samples

	**CD4+ T cells**	**CD8+ T cells**	**Treg cells**	**B cells**	**Neutro-phils**	**Total lymphocytes**	**Macro-phage**	**TLS**	**VEGFR2 expression**	**PD-1 expression**
pre-infection	0	9	2	3	0	40	8	rare	negative	negative
post-infection	0	35	0	0	1	35	5	none	negative	negative

Cell marker used for immunohistochemistry assays: Treg Foxp3, Total lymphocytes LCA, B cells CD20, Macrophage CD68.

TLS tertiary lymph node **LCA** leucocyte common antigen
