# Supplementary material for: Exceptional response to PD-1 inhibition immunotherapy in advanced metastatic osteosarcoma with tumor site infection
Source: J Immunother Cancer. 2022 Sep 9;10(9):e004673. doi: 10.1136/jitc-2022-004673 (PMC9472102; doi:10.1136/jitc-2022-004673)
Supplement: Supplementary data [file jitc-2022-004673supp005.pdf]

Supplementary Method

Luminex immunoassay of 48 inflammatory factors

Quantification of inflammatory factors was performed by Universal Biotech (Shanghai, China) using 48-Plex Bio-Plex Pro Human Cytokine Screening Panel (#12007283, Bio-rad laboratories, USA) and Luminex instrument platform (ThermoFisher, USA) according to the manufacturer's protocol. Briefly, tissue lysate was diluted and prepared in 2ug/ul before use. The magnetic beads were diluted in 10X and vortexed for 30 seconds before adding to the plate well in 50 ul. Antigen standard (50 ul) and tissue lysate (50 ul) were added to the plate and incubated for 30 minutes with 800 rpm shaking at room temperature. Then, 25 ul of detection antibody mix diluted in 10X was added to the plate, followed by incubating 50 ul of Streptavidin-PE (100X) for 30 minutes. After bead re-suspending with 125 ul Assay Buffer, data were acquired on the Luminex platform and the readouts were transformed to the original tissue concentration for further analysis.

Immune infiltration estimation by immunohistochemistry

Tissue preparation, hematoxylin-eosin stain, immunohistochemistry and pathological evaluation were performed in the pathology department of the researcher's institution. Formalin-fixed paraffin-embedding samples were stained with CD8 (Dako, Agilent, USA), CD4 (Dako, Agilent, USA), Foxp3 (Abcam, USA), CD20 (Dako, Agilent, USA), LCA (Dako, Agilent, USA), CD68 (Dako, Agilent, USA), VEGFR2 (Dako, Agilent, USA), PD-1 (ZSGB-BIO, China) and PD-L1 (Dako, Agilent, USA) to label different cell components within the tumor. According to previous reports[1 2], the density of immune cell infiltration was interpreted by a single experienced pathologist (Prof. Chen. XY) who counted the number of marker-positive cells per high-power field (40X objective, 0.55 mm in diameter), followed by averaging five random regions within the tumor area.

Microbial metagenomic sequencing

The microbial metagenomic sequencing study was performed in a well-validated hospital-based sequencing platform[3]. Briefly, specimens were mixed with 1 g of 0.5-mm glass beads and agitated vigorously at ~3000 rpm for 30 min. The extraction of sample DNA was performed with TIANamp Micro DNA Kit (DP316, Tiangen Biotech, China) according to the manufacturer's protocols. Single-end sequencing of extracted DNA with 50 cycles was automated on the BGISEQ-50 sequencer (BGI, China). In parallel with the testing, nuclease-free water was subjected to the same pipeline as negative controls. The raw sequencing data were pre-processed by low-quality and short (< 50 nucleotides) reads removal, followed by alignment to the human reference genome hg19 with Burrows-Wheeler Aligner (BWA) (version 0.7.13-r1126). The remaining unaligned reads were then mapped to National Center Biotechnology Information (NCBI, ftp://ftp.ncbi.nlm.nih.gov/genomes/) RefSeq reference database, including 3,446 bacterial species, 4,152 viral taxa, 206 fungi, and 140 parasites.

Tumor next-generation sequencing

The genomic mutation, copy-number variation and gene expression profiles of tumor samples were investigated using whole exome (WES), low-coverage(3x) whole-genome (WGS) and whole transcriptome (RNA-Seq) sequencing. Library preparation, tumor sequencing and bioinformatic interpretation service was performed by Primbio Genes Co., Ltd (Wuhan, China) according to the manufacturer's protocols. Briefly, WGS libraries were constructed using the KAPA Hyper Prep Kits（KK8504, Roche, USA）and WES libraries were prepared with the KAPA Hyper Prep Kits (KK8504, Roche, USA), followed by exome capture with xGen Exome Research Panel v2.0 (Integrated DNA Technologies, USA) according to the manufacturer's protocols. RNA-Seq libraries were constructed with KAPA RNA HyperPrep Kit with RiboErase (KAPA # KK8481, Roche, USA) per protocols. Following the assessment of library quality and quantity, next-generation sequencing was performed to generate paired-end (2×150) data on Illumina HiSeq 2500 platform (Illumina, USA) and mapped to the reference genome hg19. The researcher manually curated the result of tumor exome mutation and copy-number results to identify the potential meaningful gene alteration related to immunotherapy. Gene expression was normalized as transcripts per million (TPM) to compare the expression of potentially targetable check-point molecules.

1. Wunder JS, Lee MJ, Nam J, et al. Osteosarcoma and soft-tissue sarcomas with an immune infiltrate express PD-L1: relation to clinical outcome and Th1 pathway activation. *Oncoimmunology* 2020;9(1):1737385. doi: 10.1080/2162402X.2020.1737385 [published Online First: 2021/01/19]
2. Keung EZ, Tsai JW, Ali AM, et al. Analysis of the immune infiltrate in undifferentiated pleomorphic sarcoma of the extremity and trunk in response to radiotherapy: Rationale for combination neoadjuvant immune checkpoint inhibition and radiotherapy. *Oncoimmunology* 2018;7(2):e1385689. doi: 10.1080/2162402X.2017.1385689 [published Online First: 2018/01/09]
3. Miao Q, Ma Y, Wang Q, et al. Microbiological Diagnostic Performance of Metagenomic Next-generation Sequencing When Applied to Clinical Practice. *Clin Infect Dis* 2018;67(suppl_2):S231-S40. doi: 10.1093/cid/ciy693 [published Online First: 2018/11/14]
